# Interaction of Tri-Cyclic Nucleobase Analogs with Enzymes of Purine Metabolism: Xanthine Oxidase and Purine Nucleoside Phosphorylase

**DOI:** 10.3390/ijms251910426

**Published:** 2024-09-27

**Authors:** Alicja Stachelska-Wierzchowska, Marta Narczyk, Jacek Wierzchowski, Agnieszka Bzowska, Beata Wielgus-Kutrowska

**Affiliations:** 1 Department of Physics and Biophysics, Faculty of Food Sciences, University of Warmia and Mazury in Olsztyn, 4 Oczapowskiego St., PL-10-719 Olsztyn, Poland; alicja.stachelska@uwm.edu.pl (A.S.-W.); jacek.wie@uwm.edu.pl (J.W.); 2 Division of Biophysics, Institute of Experimental Physics, Faculty of Physics, University of Warsaw, ul. Pasteura 5, PL-02-093 Warsaw, Poland; marta.narczyk@fuw.edu.pl

**Keywords:** purine nucleoside phosphorylase, xanthine oxidase, etheno derivatives of 2-aminopurine, fluorescence, X-ray crystallography, enzyme–ligand complex, calorimetry

## Abstract

Fluorescent markers play important roles in spectroscopic and microscopic research techniques and are broadly used in basic and applied sciences. We have obtained markers with fluorescent properties, two etheno derivatives of 2-aminopurine, as follows: 1,N^2^-etheno-2-aminopurine (1,N^2^-ε2APu, **I**) and N^2^,3-etheno-2-aminopurine (N^2^,3-ε2APu, **II**). In the present paper, we investigate their interaction with two key enzymes of purine metabolism, purine nucleoside phosphorylase (PNP), and xanthine oxidase (XO), using diffraction of X-rays on protein crystals, isothermal titration calorimetry, and fluorescence spectroscopy. Crystals were obtained and structures were solved for WT PNP and D204N-PNP mutant in a complex with N^2^,3-ε2APu (**II**). In the case of WT PNP—1,N^2^-ε2APu (**I**) complex, the electron density corresponding to the ligand could not be identified in the active site. Small electron density bobbles may indicate that the ligand binds to the active site of a small number of molecules. On the basis of spectroscopic studies in solution, we found that, in contrast to PNP, 1,N^2^-ε2APu (**I**) is the ligand with better affinity to XO. Enzymatic oxidation of (**I**) leads to a marked increase in fluorescence near 400 nm. Hence, we have developed a new method to determine XO activity in biological material, particularly suitable for milk analysis.

## 1. Introduction

Fluorescent nucleoside and nucleobase analogs are tools commonly used to study the structure and dynamics of proteins, nucleic acids, and their complexes [1,2,3,4,5,6,7,8,9,10,11,12]; they are also utilized as reporters of enzymatic activity in biological material [13,14]. Tri-cyclic purine nucleoside analogs, and particularly the etheno derivates of natural purines, are among the oldest compounds used as fluorescent probes in enzymology [1,2,3]. These compounds have also been reported to feature interesting biochemical and pharmacological activities, recently reviewed by Boryski et al. [5,6]. Although they cannot be classified as isomorphic probes, they are, nevertheless, active as substrates and co-substrates of several purine-related enzymes, including ATP-ases and dehydrogenases [1].

Previously, we reported several rather unexpected activity patterns of fluorescent etheno derivatives of purines towards various forms of purine nucleoside phosphorylase (PNP, E.C. 2.4.2.1), an enzyme of the purine salvage pathway [15,16,17]. PNP is a ubiquitous enzyme that reversibly converts purine nucleosides into nucleobases and α-pentose-1-phosphate, and is responsible for the regulation of nucleoside concentration within cells [18]. This enzyme is a target of several pharmaceutical interventions and its inhibitors were shown to have a strong therapeutic effect against viral, bacterial, and parasitic infections, as well as lymphomas; they also present marked immunosuppressing activity, applicable in organ transplantations [18,19,20,21,22,23].

Recently, we have described two isomeric and fluorescent etheno derivatives of 2-aminopurine (Scheme 1) and demonstrated their potential applicability as indicators of purine metabolism enzyme activity [17,24].

2-Aminopurine readily reacted with aqueous chloroacetaldehyde to give the following two products: “linear” 1,N^2^-etheno-2-aminopurine (1,N^2^-ε2APu, I) and “non-linear” N^2^,3-etheno-2-aminopurine (N^2^,3-ε2APu, II, see Scheme 1). Spectroscopic properties of these derivatives, especially quantum yield of fluorescence (Table 1), are good and make them promising candidates to become fluorescent probes of enzymatic reactions related to purine metabolism.

These compounds were readily ribosylated by bacterial (*E. coli*) PNP in a phosphate-free media with α-ribose-1-phosphate (R1P) as a ribose donor, and the “non-linear” II reacted also with mammalian PNP (from human erythrocytes or calf spleen). The products of the enzymatic ribosylation differed spectrally from those previously known from the data from the literature [25], and the NMR analysis indicated N(2) as the main ribosylation site(s) [17].

We have purified the non-typical N(2)-riboside of the strongly fluorescent N^2^,3-ε2APu and utilized it to fluorometrically detect PNP activity in human blood [24]. It was shown that such activity is readily detectable even in 1000-fold diluted hemolysates.

During control experiments, we also realized that the less fluorescent, “linear” 2-aminopurine analog, 1,N^2^-ε2APu (I), may be a good fluorescent indicator of the activity of xanthine oxidase (XO, E.C. 1.17.3.2), an extra-cellular enzyme responsible for oxidation of hypoxanthine to xanthine with subsequent production of uric acid, which is the final step in purine catabolism [26,27]. XO is recognized as the primary target in the treatment of gout [28], but also as a factor affecting the therapeutic effectiveness of some nucleobases used as drugs, including anti-cancer pharmaceuticals like 6-thiopurine and 6-thioguanine [29,30]. This enzyme is also essential in the area of nitrite biochemistry [31]. Increased activity of XO in blood serum is an indication of liver malfunction [32]. Other medical roles of XO in humans have been recently postulated; in particular, the inhibition of xanthine oxidase, an enzyme releasing hydrogen peroxide, has been proposed as a tool for improving cardiovascular health [33,34], and inhibitors of XO were considered as drugs that could prevent the “cytokine storm” during the SARS-CoV-2 infections [35].

In the present paper, the above-described investigations were extended to include calorimetric and structural studies of PNP complexes with 1,N^2^-ε2APu (I) and N^2^,3-ε2APu (II). We chose the wild-type PNP (WT-PNP) and a D204N-PNP mutant. Mutation of Asp into Asn is a substitution of one polar amino acid with another polar amino acid having different properties, namely different pK_a_ for protonation. This mutation still allows for the formation of a hydrogen bond between a ligand and the protein, but proton transfer from the enzyme molecule to the purine ring of a substrate is impossible; thus, this mutation gives insight into the mechanism of catalysis.

Additionally, we explored the kinetic and structural aspects of XO interaction with fluorescent ligands. The aim of the presented work was to elucidate kinetic, structural, and mechanistic aspects of these non-typical enzymatic activities of PNP and XO and to present their possible applications.

The crystal structure of bacterial PNP complexed with ligands is known [36,37], but to the best of our knowledge, no tri-cyclic substrate was analyzed this way. Here, we present the structure of crystals of *E. coli* PNP with the “non-linear” substrate (II) and analyze its possible mechanism of activity.

To check the interaction of 1,N^2^-ε2APu (I) and N^2^,3-ε2APu (II) with xanthine oxidase, spectroscopic and calorimetric measurements were performed. They showed that 1,N^2^-ε2APu (I) proved to be a much better substrate for XO than the isomeric II, with reaction rates comparable to those of the natural substrates, xanthine and hypoxanthine, and affinities in the low micromolar region. The kinetic parameters allowed direct applicability of the XO assay based on 1,N^2^-ε2APu (I) oxidation to the analysis of bovine milk. XO activity in milk reflects the “thermal history” of the milk, or milk-derived samples, since this enzyme is relatively sensitive to elevated temperatures, applied during the pasteurization process [38]. This feature is important for quality control.

Attempts to obtain XO crystals in complex with etheno derivatives were unsuccessful, despite the fact that the conditions described in [39,40] were mirrored and modifications were introduced. Nevertheless, calorimetric data confirmed the formation of tight complexes between both tri-cyclic substrates and XO.

The results of our study allow for a better understanding of the properties of purine metabolism enzymes and help to determine the feasibility of using fluorescent etheno derivatives of 2-aminopurine base as markers of the activity of these enzymes.

## 2. Results

### 2.1. Purine Nucleoside Phosphorylase

**Scheme 2:**
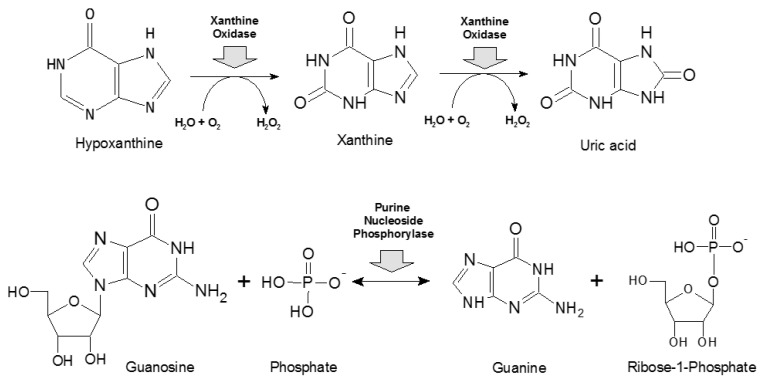
Basic reactions catalyzed by xanthine oxidase (upper panel, [41]) and purine nucleoside phosphorylase (lower panel, [18]).

PNP catalyzes reversible phosphorolysis of purine nucleoside as shown in Scheme 2. The properties of two PNP variants (trimeric and hexameric) are described in review papers [18].

#### 2.1.1. Interaction of WT PNP and D204N-PNP with Etheno Derivatives of 2APu

In the previous paper [17], we described the substrate properties of two fluorescent etheno derivatives of 2-aminopurine: N^2^,3-ε2APu and 1,N^2^-ε2APu. Only N^2^,3-ε2APu is a substrate for the enzyme from a calf spleen, while both compounds are ribosylated using R1P as a ribose donor and bacterial *E. coli* PNP as a catalyst. 1,N^2^-ε2APu reacts slowly with K_m_ < 10 μM and relative V_max(rel)_~1 (relative to guanine ribosylation rate, V_max(rel)_ = 100, under identical conditions), while N^2^,3-ε2APu reacts rapidly (K_m_ = 12 μM, V_max(rel)_ = 20). When the mutant D204N-PNP with Asp204 replaced by Asn204 was chosen as a catalyst, the obtained data for N^2^,3-ε2APu was similar to the WT PNP (K_m_ = 12 μM, V_max(rel)_ = 30).

Analysis of UV and NMR spectra of products led to the conclusion that, in all cases, the predominant ribosylation site is N2, rather than N9, yielding 1,N^2^-ε2APu-N^2^-β-D-riboside or N^2^,3-ε2APu- N^2^-β-D-riboside, respectively [17].

To investigate the cause of such non-canonical ribosylation, we crystallized the following complexes of *E. coli* PNP with etheno derivatives of 2-aminopurine: WT PNP-N^2^,3-ε2APu, WT PNP-1,N^2^-ε2APu, D204N-PNP-N^2^,3-ε2APu, and D204N-PNP-1,N^2^-ε2Apu, and solved their structures.

**Figure 1:**
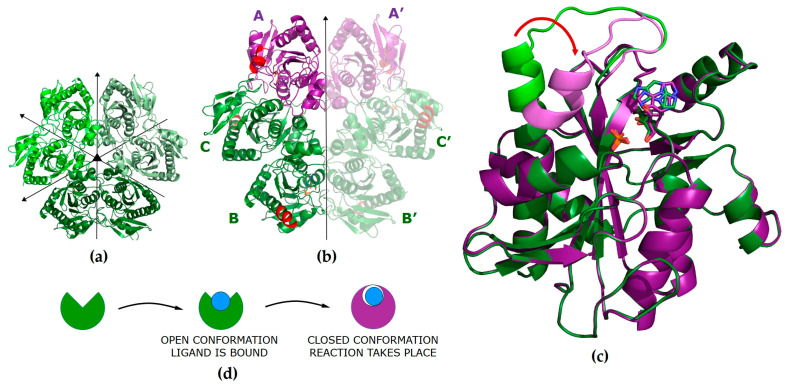
(**a**) Model of the WT PNP hexamer in the apo form (PDB 1ECP [42]) in the P12_1_1 space group with all subunits in the open active site conformation. Symmetries of the molecule are denoted with arrows. (**b**) Model of the WT PNP molecule in a complex with N^2^,3-ε2APu in the P6_1_22 space group (PDB ID 9FPE). The asymmetric unit contains half of the hexamer (subunits A, B, C). The subunits in the closed active site conformation are shown in violet. Part of the helix H8 (amino acids 214–236) that changes conformation upon ligand binding and closes the entrance to the active site [37] is shown in red. (**c**) Comparison of the monomers in the open and closed active site conformations. Direction of the helix H8 movement is shown by the red arrow. (**d**) A scheme showing conformational changes of the PNP subunit occurring during the process of ligand binding and catalysis. In the open conformation, the ligand is bound, while in the closed conformation, catalysis is performed.

All obtained protein–ligand complexes crystallized in the space group P6_1_22, with half of the hexamer in the asymmetric unit (3 monomers marked A, B, C). The active hexamer is formed by the symmetry operations (two-fold axis). Dimers forming a hexamer are marked A-C, A’-C’, and B-B’, where ’ denotes monomers from the adjacent asymmetric unit (Figure 1b).

We introduced the concept of a closed and open conformation of active sites of this enzyme and their role in catalysis in [36]. The hexameric molecule of PNP from *E. coli* can be described as a trimer of dimers (Figure 1a). In the apo form, all monomers are in the open conformation, but upon phosphate ion (one of the substrates) binding, part of them adopts a closed conformation, forming asymmetrical open-closed dimers (Figure 1b, violet/green subunits indicates closed/open conformation, respectively). In the open conformation, helix H8 (amino acids 214–236) forms a single continuous segment, leaving the entrance to the active site pocket wide open and resulting in the loose binding of ligands. However, in the closed conformation, helix H8 is divided into two segments (H8: 214–219 and H8’: 223–236), separated by a γ-turn, with the H8 part of the helix moved towards the entrance of the active site, partially closing it and resulting in the ligands being bound more tightly (Figure 1c).

The key role in the catalysis of natural enzyme substrates is played by Asp204, which is protonated, and by Arg217. In the open conformation, the ligands are bound, and Asp204 forms a hydrogen bond with N7 of the purine ring, but due to a large distance between Asp204 and Arg217, the reaction does not take place. Closing of the active site is a necessary step in catalysis; it brings Arg217 closer to Asp204 and enables the formation of a hydrogen bond between these residues, with deprotonation of Asp204 and protonation of N7 of the purine ring, which initiates catalysis.

#### 2.1.2. Interaction of 1,N^2^-ε2APu with WT PNP and D204N-PNP from X-ray Diffraction

**Figure 2:**
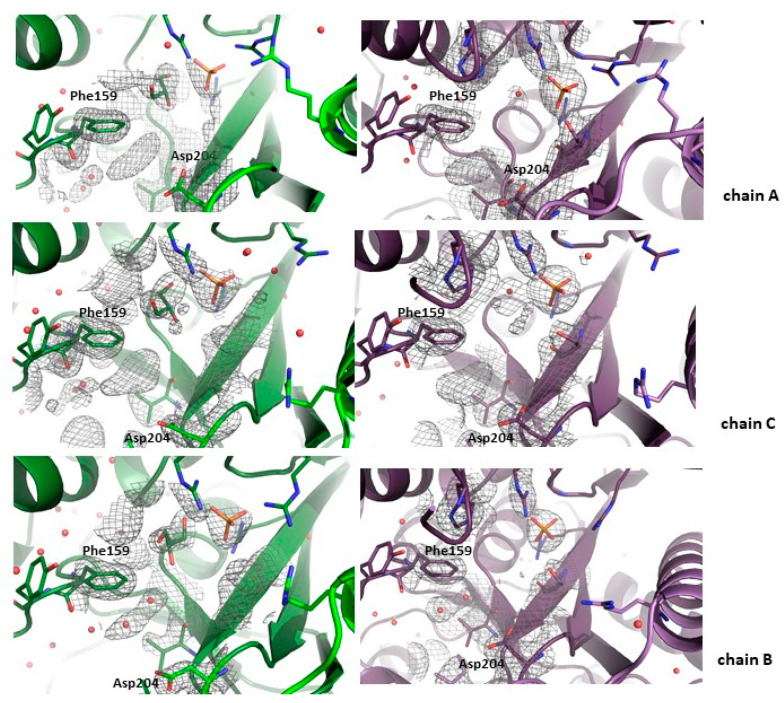
Active sites of the WT (green) and the D204N mutant (violet) of *E. coli* PNP co-crystallized with 1,N^2^-ε2APu. Omit electron density maps mFo-DFc are shown at 3σ level. In all subunits, active sites are in the open conformation. In both proteins in subunits A and C, belonging to one dimer, some faint electron density blobs are visible below Phe159 side chain, in the place usually occupied by the purine ring of natural nucleoside substrates. These blobs are, however, too small to fit 1,N^2^-ε2APu in them, yet their shape suggests that in some small fraction of enzyme molecules, the ligand is probably bound. In chains B in both protein variants, only water molecules are found in this region of the active sites.

In the structures of WT PNP-1,N^2^-ε2APu and D204N-PNP-1,N^2^-ε2APu, the ligand molecule could not be modeled in any of the active sites, despite the fact that the electron density blobs are present in all active sites close to the Asp204 or Asn204 side chains, respectively. Both structures, WT PNP and D204N-PNP, are very similar and thus can be described together. In both complexes, only the open active site conformation is observed. A phosphate ion (or a sulfate ion from the crystallization liquid) is bound in all of them, in a typical position, where it can form hydrogen bonds with Arg43* (from a neighboring subunit in a dimer) and Arg87, whereas Arg24 points away from the active site. In subunit B of both complexes, in the place usually occupied by the purine ring, there is an array of small electron density blobs fitting well with few water molecules. In subunits A and C (forming one dimer), blobs have shapes similar to the tested ligand but they are too small to fit in a 1,N^2^-ε2APu molecule (Figure 2). This most likely indicates that the ligand binds only in a small number of protein molecules and may explain the low V_max(rel)_ of enzyme reaction previously obtained in kinetic measurements [24].

#### 2.1.3. Interaction of N^2^,3-ε2APu with WT PNP and D204N-PNP from X-ray Diffraction

**Figure 3:**
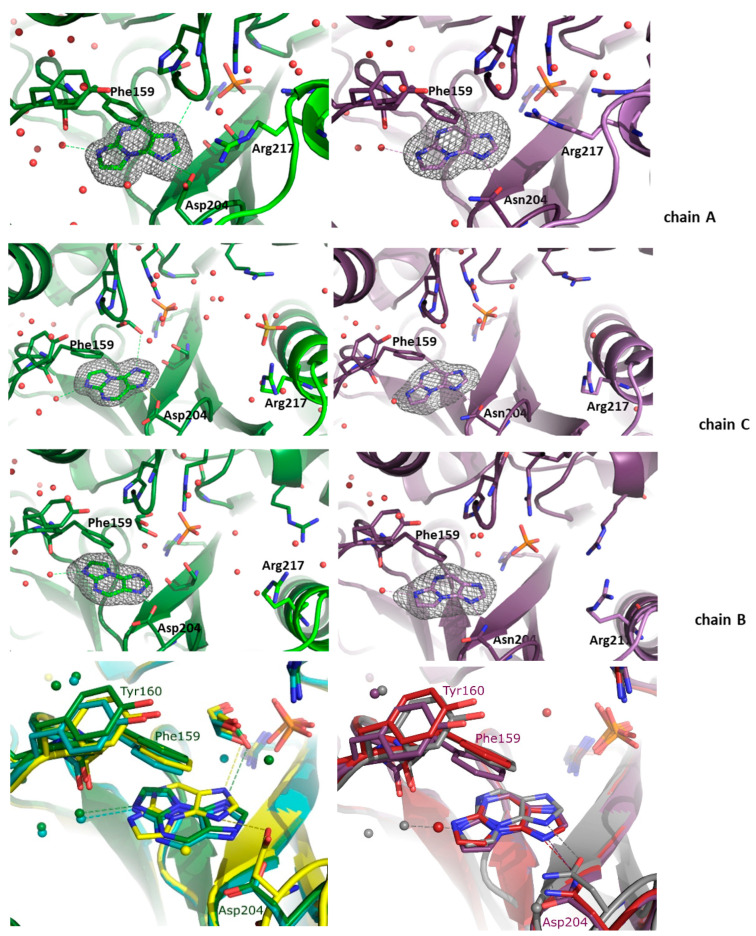
Active sites of the WT (green, left panels) and D204N mutant (violet, right panels) of *E. coli* PNP hexamer complexes with N^2^,3-ε2APu. Omit electron density maps mFo-DFc at 3σ level are shown only around this ligand. In both structures, the active site of chain A is in the closed active site conformation, and chains B and C are in the open active site conformation. In all subunits of both protein variants, N^2^,3-ε2APu is clearly visible in the electron density map. But as the ligand and the electron density blob are symmetric, orientation of the ligand molecule cannot be unequivocally determined. Since in the case of natural substrates, interaction of the substrate with Asp/Asn 204 is crucial for catalysis, here, the ligand is shown in the orientation allowing formation of a hydrogen bond with Asp/Asn204 (both possible orientations and their consequences for catalysis are shown in Scheme 3 and discussed in the text). Bottom panels: aligned structures of the WT-PNP (left panel) and the D204N-PNP (right panel) complexes with N^2^,3-ε2APu. WT enzyme: subunit A (closed) is yellow, subunit C (open, from the AC dimer) is green, subunit B (open) is cyan. D204N mutant: subunit A is gray, subunit C is red, subunit B is violet.

In the WT PNP-N^2^,3-ε2APu and D204N-PNP-N^2^,3-ε2APu structures, the ligand is bound in all subunits, as the electron density in the appropriate region of the active site is in this case unambiguous (Figure 3). In both structures, there is one subunit in the closed (chain A) and two subunits in the open (chains B and C) active site conformations; thus, an active hexamer is composed of two asymmetric open–closed dimers (A-C, A’-C’) and one symmetric open–open dimer (B-B’). In all active sites, a phosphate ion (or a sulfate ion from the crystallization liquid) is bound in a typical position, at a hydrogen bond distance from Arg43* (from the neighboring subunit) and Arg87, while in the active sites with the closed conformation, also from Arg24. As there is no pentose in the N^2^,3-ε2APu molecule, in the case of the WT enzyme, this part of the active site is occupied by glycerol, which was used as a cryoprotectant. In the case of the D204N enzyme, small electron density blobs observed in this part of the active site were modeled with a few water molecules. The orientation of N^2^,3-ε2APu in the active site cannot be determined unambiguously as, at this resolution, the electron density blob is symmetric.

In the case of the WT PNP, the orientation differs in the closed and open active site conformations, whereas in the structure of the D204N enzyme, the orientation is identical in all active sites (Figure 3).

In the closed active site conformation of the WT complex, N^2^,3-ε2APu can be modeled in an orientation where a hydrogen bond between Asp204 and N9 of the ligand is possible but, in this orientation, ribosylation would occur at N7. In the second possible orientation, C11 of the ligand is at a hydrogen bond distance from Asp204; thus, the hydrogen bond cannot be created, but ribosylation would occur on N^2^, which, indeed, predominantly occurs as described in Section 2.1.1 (see also Scheme 3a,b).

For the WT PNP-N^2^,3-ε2APu complex, in the active sites with the open conformation, the ligand is “flipped” (Figure 3 and see also Scheme 3c,d). In principle, the ligand can be again fitted into the electron density blob in two orientations. In one of them, a hydrogen bond can be created between Asp204 and N7 of the ligand; thus, ribosylation would occur on N9, while in the second one, N^2^ would be at a hydrogen-bonding distance but then ribosylation would occur on C11, which is impossible.

In the case of the D204N-PNP-N^2^,3-ε2APu complex, the orientation of the ligand in all subunits, regardless of the active site conformation, is like in the closed active site of the WT PNP, thus pointing to the ribosylation site either at N7 or N^2^.

#### 2.1.4. Interaction of 1,N^2^-ε2APu and N^2^,3-ε2APu with WT PNP by Isothermal Titration Calorimetry

**Figure 4:**
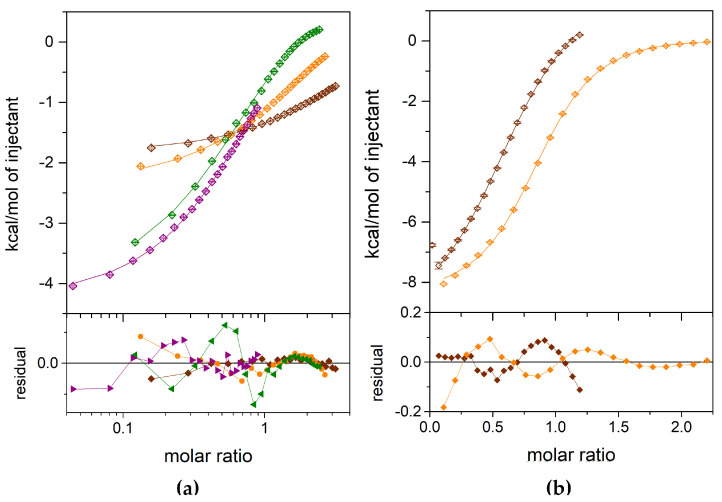
Calorimetric titrations of the *E. coli* WT PNP with 1,N^2^-ε2APu (**a**) and N^2^,3-ε2APu (**b**). Data (points), fits of a one-site model (lines) and residuals (lower graphs) are shown. Titrations were performed with the following concentration of the protein in the cell: for 1,N^2^-ε2APu (panel **a**), 348.33 µM (brown), 1198.39 µM (orange), 3543.87 µM (green), 3484.83 µM (violet); for N^2^,3-ε2APu (panel **b**), 253.86 µM (brown), 474.24 µM (orange).

To check why the 1,N^2^-ε2APu (**I**) reacts, albeit slowly, with *E. coli* PNP, yet is not clearly observed in the crystallographic structure of the enzyme, we performed calorimetric titrations of the enzyme with both etheno derivatives of 2-amino purine. The results are presented in Figure 4. Several independent titrations performed for each ligand were analyzed globally using the ITCsy (version 1a) software. This method allows fitting simultaneously and directly one global model to all experimental data, and can provide significantly more detailed and precise information about molecular interactions, binding affinity, and enthalpy than other programs [43].

For both ligands, the one-binding-site model is sufficient to describe the obtained ITC data (Figure 4). An attempt to fit a different, two-site model failed, because, in the case of the N^2^,3-ε2APu, no fit convergence was obtained, while for the 1,N^2^-ε2APu, the discrepancies between the data and the fitted curve are greater than those for the one-binding-site model.

Finally, for the N^2^,3-ε2APu (**II**), we obtained K_d_ = 36.16 μM, with the asymmetric confidence intervals (29.33–45.89) μM, while for 1,N^2^-ε2APu, K_d_ is larger than 3.5 mM, c.a. 100 times, as that observed for the N^2^,3-ε2APu.

### 2.2. Xanthine Oxidase

#### 2.2.1. Attempt to Crystallize Xanthine Oxidase

**Figure 5:**
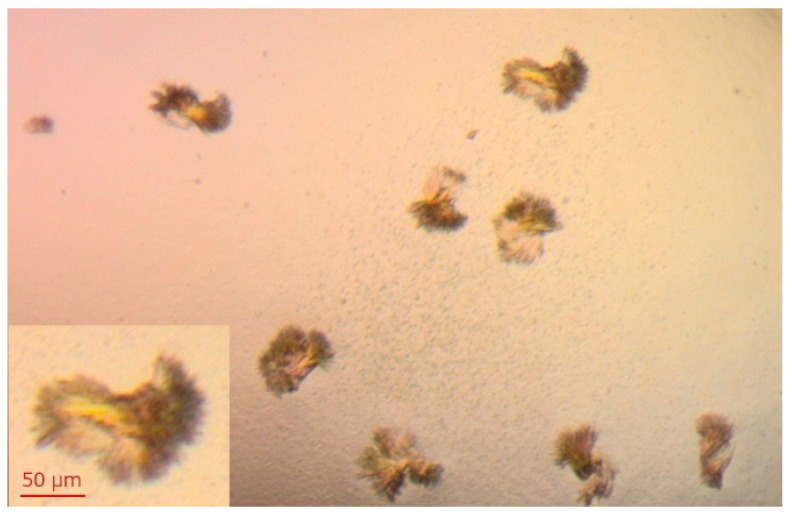
Crystals of XO obtained by mixing 2 μL of 15 mg/mL XO + 5 mM DTT with 2 μL of crystallization solution (14% PEG 8000, 0.2 mM EDTA, 100 mM potassium phosphate buffer pH 6.5). Crystallization was carried out at 18 °C using the hanging-drop diffusion method. Similar crystals were obtained in a few drops of varying protein concentration (7.5–15 mg/mL). The protein was crystallized in the apo form, and soaking crystals with the ligands solution was planned.

Xanthine oxidase is the second enzyme of the purine metabolism for which we decided to check the substrate activity of 1,N^2^-ε2APu, and N^2^,3-ε2APu, and details are described in the following sections. As for PNP, we also tried to characterize interactions in solution by isothermal titration calorimetry and the structure of complexes by X-ray studies. However, despite a number of crystallization conditions screened, based on the data from the literature [39,40], we have so far been unable to obtain crystals that scatter X-rays with a resolution good enough to obtain the structure of the complexes. The best crystals obtained are presented in Figure 5.

#### 2.2.2. Isothermal Titration Calorimetry

**Figure 6:**
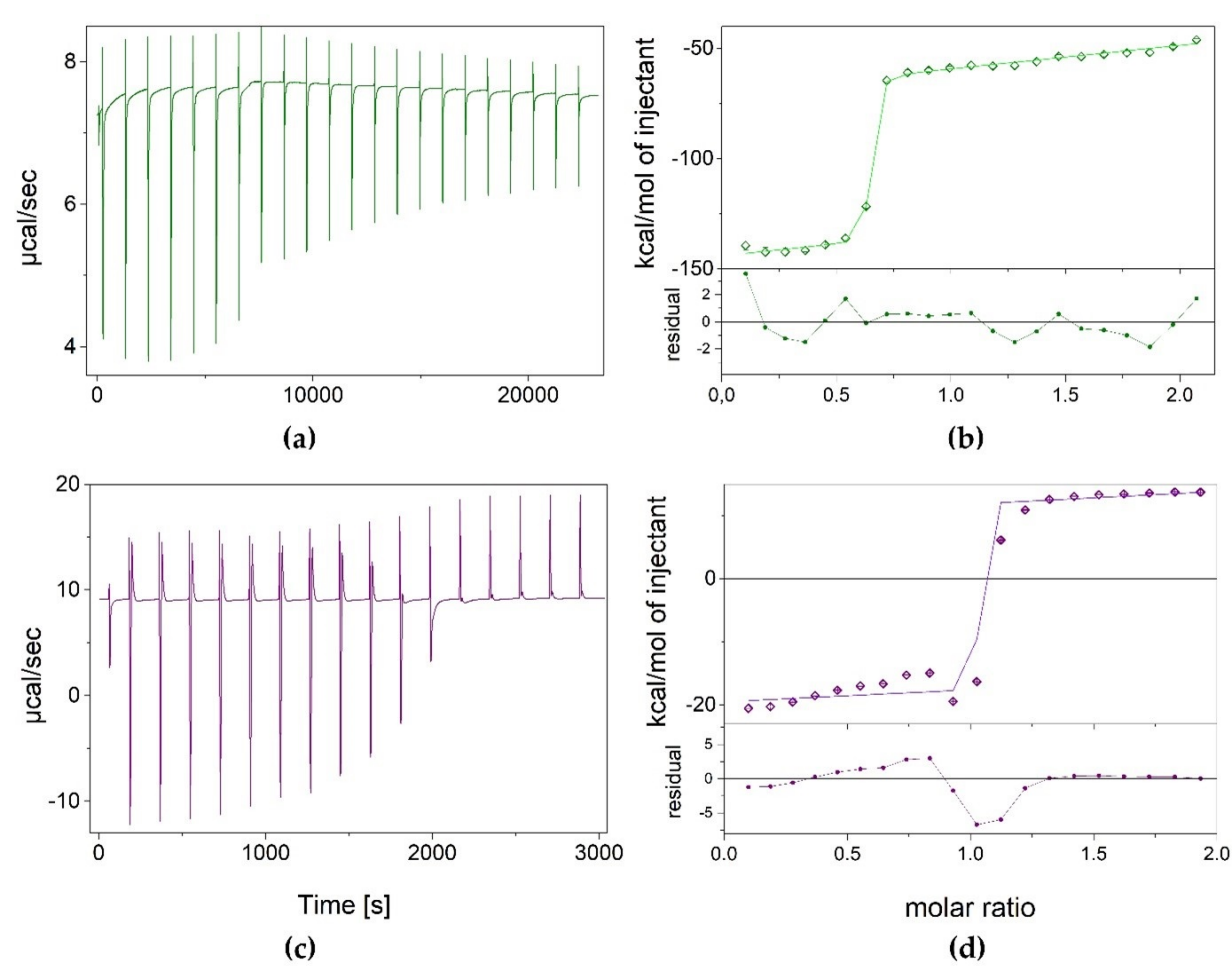
Results of isothermal calorimetric titrations of XO with N^2^,3-ε2APu (green) and 1,N^2^-ε2APu (violet), thermograms (panels **a**,**c**), and isotherms (panels **b**,**d**). Experimental data (points), curves of the fitted one-binding-site model (lines) as well as residual plots are shown. Titrations were performed at 25 °C, in 50 mM potassium phosphate buffer with 1 mM TCEP pH 8.0, with 84.04 μM and 458.42 μM XO in the cell for N^2^,3-ε2APu and 1,N^2^-ε2APu titrations, respectively.

As N^2^,3-ε2APu (**II**) binding to XO was not accompanied by a clear change in fluorescence and we were not able to confirm ligand–enzyme binding using other methods (crystallography, fluorescence titrations), we decided to use isothermal titration calorimetry to characterize interactions of both compounds with XO. The obtained results are presented in Figure 6.

Measurements for 1,N^2^-ε2APu (**I**) were performed at a protein concentration of 458.42 μM. It appears that the dissociation constant is very low, c.a. 2.2 nM, as estimated by fitting the one-binding-site model (Figure 6), which indicates strong binding. However, the ligand binding process seems to be complicated, and consists of both exo- and endo-thermal steps, making it difficult to interpret these data.

Although measurements for N^2^,3-ε2APu (**II**) were performed at a lower enzyme concentration (~80 μM), the situation looks similar, namely the endo- and exothermic processes were again observed. The K_d_ value was estimated at 7.5 nM.

Although calorimetric titrations were repeated many times, with increasingly longer intervals between injections, even up to 15 min, the system did not reach equilibrium, suggesting the existence of some other very slow process accompanying the binding. In addition, as K_d_ values are so small, we expected to obtain better titration curves with significantly lower protein concentration in the calorimetric cell, but surprisingly, the binding is not visible in those conditions either (see exemplary data in Supplementary Data S1).

Thus, it can be seen that both ligands bind to the enzyme, but the obtained dissociation constants are estimates only. Therefore, we decided to focus on detailed characterization of the properties of the ligands as substrates of xanthine oxidase.

#### 2.2.3. Purified Enzyme Reactions—Spectral Effects and Reaction Kinetics

**Scheme 1:**
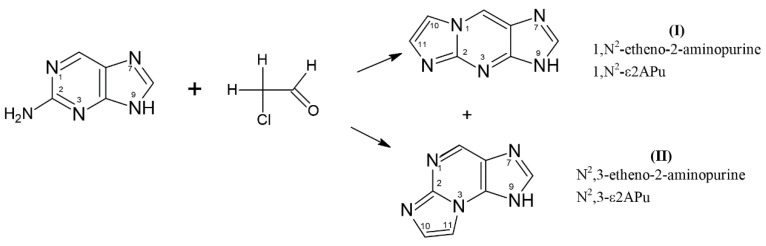
Generation of tri-cyclic etheno derivatives of 2-aminopurine base: 1,N^2^-etheno-2-aminopurine (1,N^2^-ε2APu, **I**) and N^2^,3-etheno-2-aminopurine (N^2^,3-ε2APu, **II**) in the presence of aqueous chloroacetic aldehyde. Only one tautomeric form is given for simplicity.

**Figure 7:**
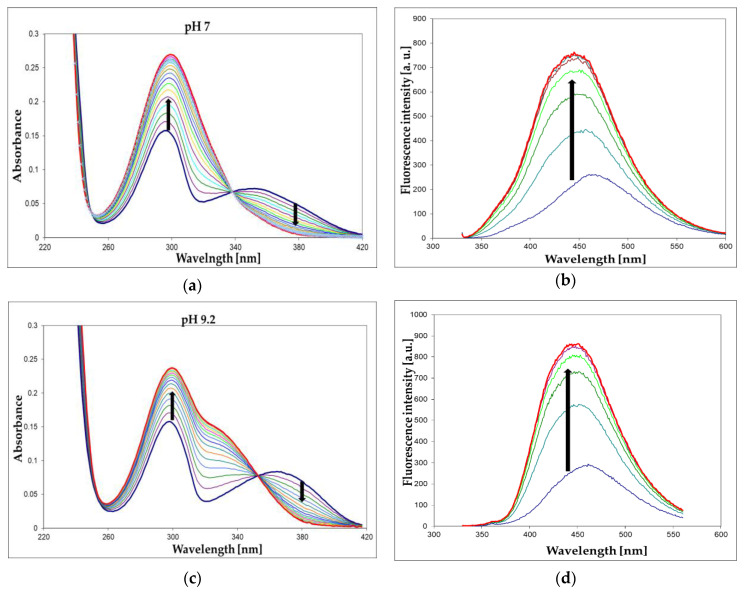
UV absorption (**a**,**c**) and fluorescence changes (**b**,**d**) observed during the enzymatic oxidation of 1,N^2^-ε2Apu (**I**) at pH 7 (**a**,**b**) and 9.2 (**c**,**d**). Fluorescence was excited at 325 nm for pH 7 and at 320 nm for pH 9.2 measurements. Concentrations: ca. 30 μM for absorption and ca. 10 μM for fluorescence detection method; temperature was 25 °C. Time intervals were 2 min (absorption) or 5 min (fluorescence). Black arrows indicate the direction of spectra changes during the reaction. Enzyme (commercial XO from bovine milk) concentration was ca. 0.001 mg/mL.

**Figure 8:**
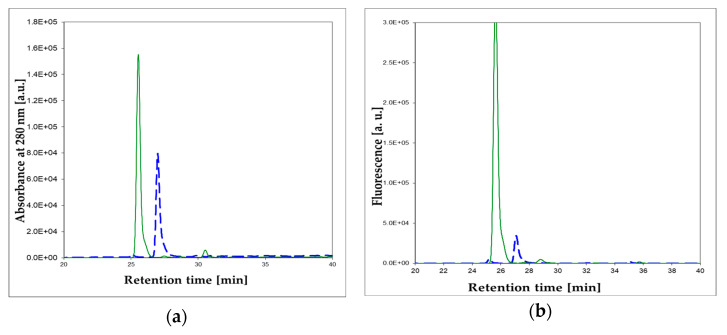
Enzymatic oxidation, catalyzed by the commercial XO from bovine milk, of 1,N^2^-ε2APu (**I**). HPLC analysis of the reaction mixture: (**a**) absorbance at 280 nm; (**b**) fluorescence at 400 nm. Dashed blue lines: prior to the reaction, solid green lines: after 1 h reaction. 1,N^2^-ε2APu (**I**) concentration was 100 μM, the commercial XO enzyme was 2000× diluted. Reaction was run at ca. 25 °C, pH 7.8.

We have screened both isomers of etheno-2-aminopurine, 1,N^2^-ε2APu (I), and N^2^,3-ε2APu (II) (Scheme 1), for activity against a commercial XO from bovine milk using absorbance as well as fluorescence detection. Of these, in contrast to PNP, 1,N^2^-ε2APu was found to be the best and also highly fluorogenic substrate (Figure 7). HPLC analysis of the reaction mixture (Figure 8) shows that essentially one product is created and no subsequent reaction(s) are observed. This is in line with the clear-cut isosbestic points observed on UV spectra (Figure 7a,c).

The enzymatic oxidation rate of (I) at pH 7 and temperature 25 °C is comparable to that of the xanthine oxidation rate in the same conditions (see Section 2.2.5). At higher concentrations (>40 μM), a marked substrate inhibition is observed for 1,N2-ε2APu (I), as documented below (Section 2.2.5), analogous to that observed for xanthine, particularly at pH 7 [44].

A highly fluorescent N^2^,3-ε2APu (**II**) isomer is only a weak substrate for bovine XO, with a reaction rate at least 10-fold slower than that observed for substrate (**I**). The main product of this reaction is also intensely fluorescent, but its emission spectrum does not differ markedly from that of the substrate (see Table 2 and Supplementary Data S4), making fluorimetric assay of the reaction difficult. We have therefore focused on the properties of 1,N^2^-ε2APu (**I**) as the optimal fluorogenic substrate of XO.

#### 2.2.4. Identification of the Product of the 1,N^2^-ε2APu Oxidation

Oxidation of 1,N^2^-ε2APu (**I**) can lead to three different products (Scheme 4).

To identify the product(s) of enzymatic oxidation of (**I**), catalyzed by the bovine XO, HPLC analysis of the final reaction mixture was performed with absorbance and fluorescence detection (see Figure 8a,b). It reveals one main and one minor (<5%) product. The elution was initially (15 min) isocratic, followed by a 10 mM phosphate buffer-methanol gradient of 1–25%. The main, intensely fluorescent product was eluted at ~27 min. By analogy with xanthine, we tentatively identify this product as (**Ia**) (Scheme 4). The minor, non-fluorescent peak at ca. 32 min has been identified as 1,N^2^-etheno-guanine (**Ib**).

Identification of the oxidation products of 1,N^2^-ε2APu (**I**) was based on the comparison of their absorption and fluorescence spectra at various pH with the reference samples described previously [16,17]. The spectral characteristics of all substrates and products are summarized in Table 2. We were able to isolate only less than 5 milligrams of the main product due to extensive substrate inhibition of the enzyme and the resultant poor yields of reactions conducted on a larger scale. The electronic spectra of the products are pH-dependent, with spectrophotometrically estimated pK_a_ values given in Table 2. At pH 7, the main product seems to be spectrally inhomogeneous, with an emission spectrum somewhat dependent on excitation wavelength. We ascribe this effect to prototropic tautomerism (probably N7H-N9H). At pH > 9, both absorption and fluorescence excitation spectra are markedly shifted towards the red, with little change in the emission spectrum. The excitation spectra are in line with the UV absorption. The fluorescence yield of the reaction product at pH 7 is 0.30–0.34, and therefore among the highest recorded for nucleobase derivatives [1,3].

#### 2.2.5. Optimization of Oxidation Reaction Conditions

The largest relative fluorescent increase during 1,N^2^-ε2APu (**I**) and N^2^,3-ε2APu (**II**) oxidation are observed for excitations at 310–320 nm and fluorescence monitored at ca. 400 nm, where ~1000% signal increase is observed for a completed reaction (see previous section).

**Figure 9:**
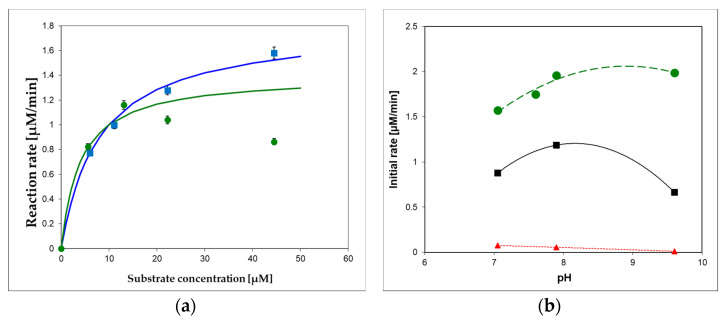
(**a**) Commercial XO from bovine milk activity in 10 mM buffers, pH 7.6 (green circles) and 9.6 (blue squares) at 30 °C, as a function of 1,N^2^-ε2APu (**I**) concentration. Michaelis–Menten curves were fitted with K_m_ = 8 μM (at pH 9.2, blue) and 4 μM (at pH 7.6, green), (**b**) pH dependence of enzymatic oxidation rates for **I** (1,N^2^-ε2APu)—green, (**II**) (N^2^,3-ε2APu)—red, and xanthine—black squares. Initial rates were measured spectrophotometrically. The commercial enzyme (~0.3 U/mL) was diluted 3000-fold.

To optimize substrate concentration and pH of the applied buffer(s) for the XO oxidation of 1,N^2^-ε2APu (**I**) and N^2^,3-ε2APu (**II**), we measured pH-activity dependence of the reaction, with results illustrated in Figure 9. As can be seen, oxidation of the optimal substrate (**I**) progresses smoothly both at pH 7 and 9.5, although the pH dependence is somewhat different for various substrates (Figure 9b). In all instances, the optimal excitation wavelength for 1,N^2^-ε2APu (**I**) is 310–320 nm, provided that substrate concentration is <20 μM (due to the inner filter effect, which can be neglected at these concentrations).

As shown in Figure 9, the oxidation reaction does not obey the Michaelis–Menten model, as previously documented for xanthine [44], but at pH 9.6, the kinetics is more regular, and the apparent K_m_, ~8 μM, can be calculated for substrate **I** (Figure 9a). At pH 7, the apparent K_m_ can be estimated to be less than 4 μM, and the maximum rate is observed at concentrations 10–15 μM (Figure 9b). Optimal substrate concentrations are therefore 10–15 μM at pH 7 and ~40 μM at pH 9.2. Preliminary estimations show that the sensitivity values at pH 7 and 9 are comparable.

#### 2.2.6. XO Activity in Milk

**Figure 10:**
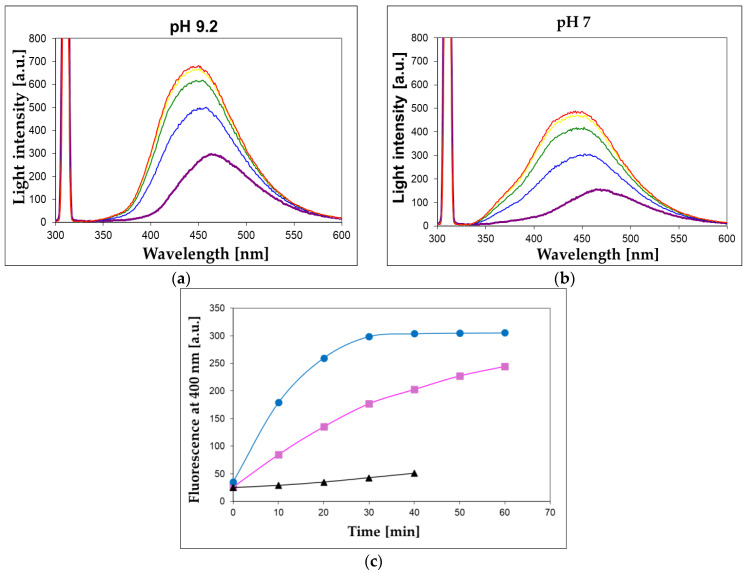
Observation of XO activity in the 100-fold diluted unprocessed milk sample at pH 7 (**a**) and 9.2 (**b**). 1,N^2^-ε2APu (**I**) concentration was 40 μM, temperature 30 °C, excitation wavelength 310 nm, time intervals between spectra recordings were 10 min. (**c**) Time evolution of the fluorescent signal at 400 nm for unprocessed (blue circles) and commercial (magenta squares) milk samples at pH 9.2. Black triangles refer to the commercial sample boiled for ca. 15 s in a microwave oven.

To check the applicability of the proposed assay involving 1,N^2^-ε2APu (**I**) and N^2^,3-ε2APu (**II**) for food analysis, we examined bovine milk samples (known to be a rich source of XO), with the results shown in Figure 10, below.

We found that XO activity is readily detectable in 100- to 200-fold-diluted bovine milk samples using the presented method utilizing 1,N^2^-ε2APu (**I**). Although the turbidity of this solution is considerable (extinction amounts to ~0.8 per 1 cm pathlength at 300 nm), the fluorescence signal is readily observable, and the reaction concludes within ~30 min (Figure 10), with the final signal remaining stable for at least 2 h. Conventional right-angle instrumental setup and semi-micro cuvettes with a 4 mm optical path for excitation pathway were used, and the scattering did not interfere with the fluorescence and the rate calculations, although the fluorescence signal was reduced by ~50% (see Methods section and Figure 10, upper panel). Reproducibility of the bovine milk assay (at pH 7, time intervals of 2 min) was better than 10% (based on 3 repeats).

The unprocessed milk was more active than the commercial samples (Figure 10c), while no activity was found in the coffee whitener or yogurt samples. The milk XO activity was fairly stable for several days, when milk was stored at 5 °C, and freezing milk for 30 min did not abolish this activity. By contrast, boiling the milk sample for 15 s reduced the XO activity by ca. 90% (Figure 10c).

The linearity check of the XO assay is presented in Appendix A (Figure A1).

#### 2.2.7. Enzyme Inhibition and Competition with Xanthine

To confirm the identity of the activity observed in milk samples as XO activity, we conducted kinetic experiments, with (**I**) as a substrate, in the presence of increased concentrations of oxipurinol, a well-known strong inhibitor of XO [45,46], or in the presence of the non-fluorescent substrate, xanthine. Better results were obtained at pH 7, where the inhibitory effect was stronger.

**Figure 11:**
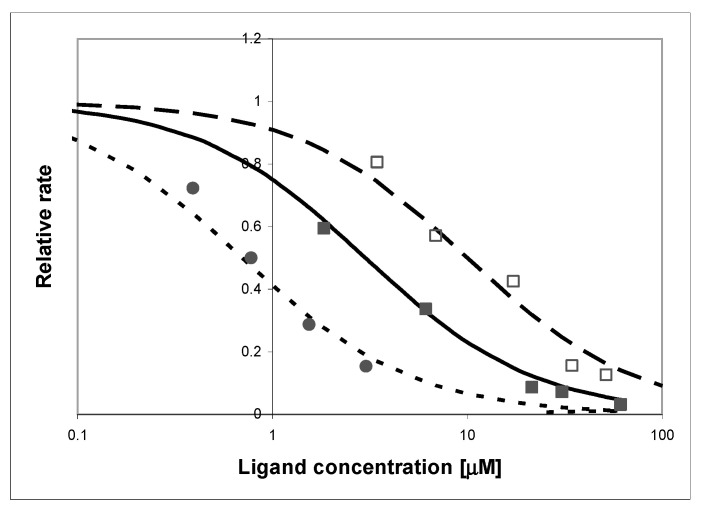
Effect of adding oxipurinol (circles) and xanthine (squares) on the rate of oxidation of (**I**) by XO, as detected by the fluorimetric procedure: (□) purified enzyme, inhibited by xanthine (as a competitor), (■) commercial milk sample, inhibited by xanthine, (●) commercial milk sample inhibited by oxipurinol. Substrate concentration was 10 μM, pH 7, temperature 30 °C. Lines refer to the apparent inhibition constants of 0.7 μM (dotted line), 3 μM (solid line), and 10 μM (dashed line).

**Figure A1:**
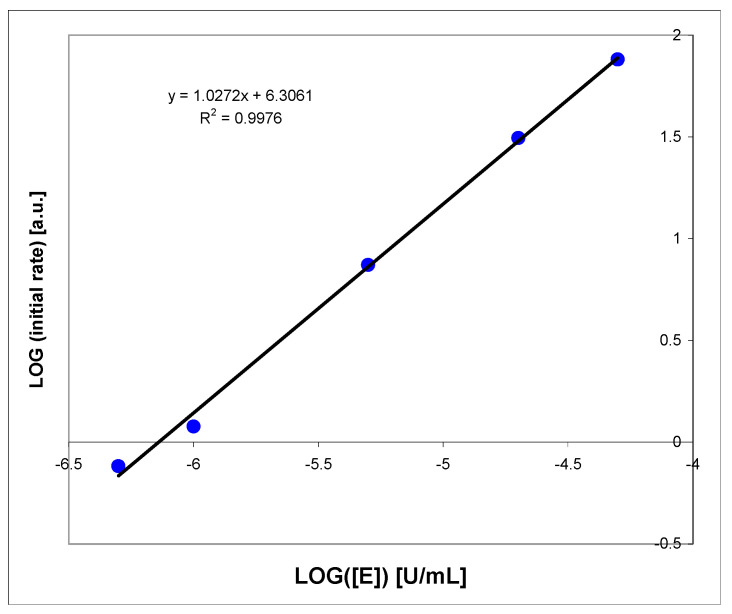
Linearity check of the oxidation rate of 1,N^2^-ε2APu (**I**) by the purified bovine milk XO, at pH 7. Substrate concentration was 10 μM, fluorescence was excited at 310 nm.

The effect of adding oxipurinol on the kinetics of the oxidation of (**I**) by 200-fold diluted milk is shown in Figure 11. As pointed out in the literature [45], this inhibition is biphasic, with the apparent inhibition constant changing from ~1.5 μM at the start to less than 0.7 μM after 15 min of the reaction (Figure 11), and milk pre-incubation with the inhibitor had little effect.

In both, the purified enzyme and milk samples, competition with xanthine was also evident, although the observed inhibitory effect was stronger for the purified enzyme (apparent inhibition constants 3 μM vs. 10 μM). The reason for this difference is not known (a possible explanation is a genetic polymorphism [27]), but the presented results indicate that the reaction is almost totally quenched by xanthine in both cases, indicating that XO is the only enzyme responsible for the fluorogenic oxidation process in bovine milk.

**Figure A2:**
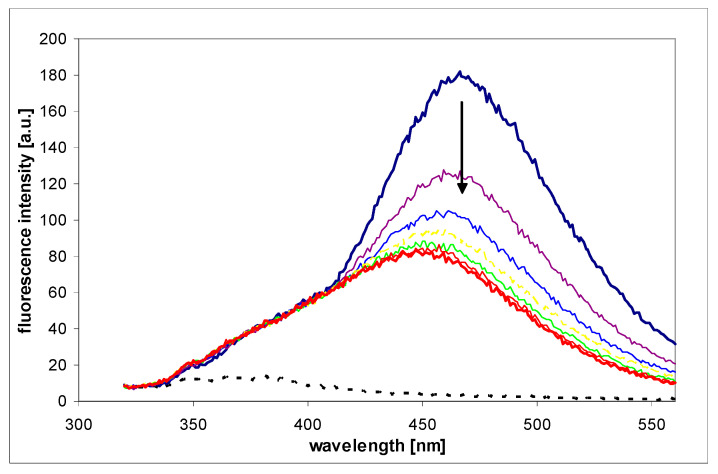
Fluorescence changes recorded during the process of oxidation of 1,N^2^-etheno-2-APu (**I**) by pork liver homogenate (see text for details). Fluorescence was excited at 310 nm, time intervals were 5 min. Homogenate background is indicated by black dotted curve, and reaction endpoint given in red.

Attempts were also made to determine oxidation of (**I**) by pork liver homogenate, but in this case, oxidation did not lead to a fluorescent product (see Appendix B, Figure A2).

HPLC analysis of the milk-catalyzed reaction is given in Supplementary Data S2.

## 3. Discussion

Fluorescent markers are important and sensitive tools for studying enzyme activity. Labels that react specifically with individual proteins are sought. Measurable fluorescence in a sample indicates the presence of active biomolecules and its intensity can give information about the concentration and/or activity of these objects.

In the present paper, we have examined two etheno derivatives of 2-aminopurine: 1,N^2^-etheno-2-aminopurine (1,N^2^-ε2APu, **I**) and N^2^,3-etheno-2-aminopurine (N^2^,3-ε2APu, **II),** both with good fluorescent properties. These compounds were previously [17] shown to exhibit substrate properties towards two key enzymes of purine metabolism—purine-nucleoside phosphorylase (PNP) and xanthine oxidase (XO)—and in the case of PNP, the riboside of (**II**) was successfully applied to detect PNP activity in human blood [24]. However, the mechanism of this interaction(s) remained unknown.

**Scheme 3:**
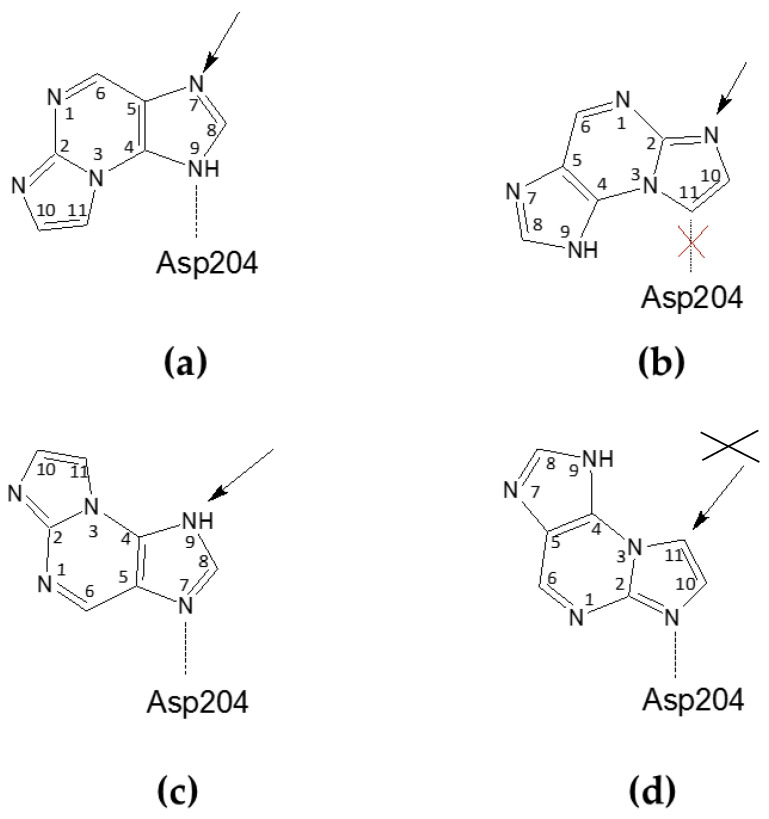
Possible orientations of the N^2^,3-ε2APu in the active site of *E. coli* PNP, relative to the catalytic Asp204 (Asn204 in the D204N mutant) residue. The arrow shows a potential glycosylation place, being a consequence of the particular orientation. (**a**,**b**) Ligand orientations consistent with the electron density observed in the closed active site conformation of the WT PNP and in the open and closed active site conformations of D204N-PNP, leading to glycosylation at N9 or N^2^, respectively; however in the orientation shown in (**b**), the hydrogen bond between Asp204 and the ligand is not possible. (**c**,**d**) Ligand orientation is consistent with the electron density observed in the open active site conformation of WT PNP, leading to potential glycosylation at N9 and C11, respectively; however, glycosylation at C11 is not possible and, as in this case, the C atom (not the N atom) would be the glycosylation site.

**Scheme 4:**
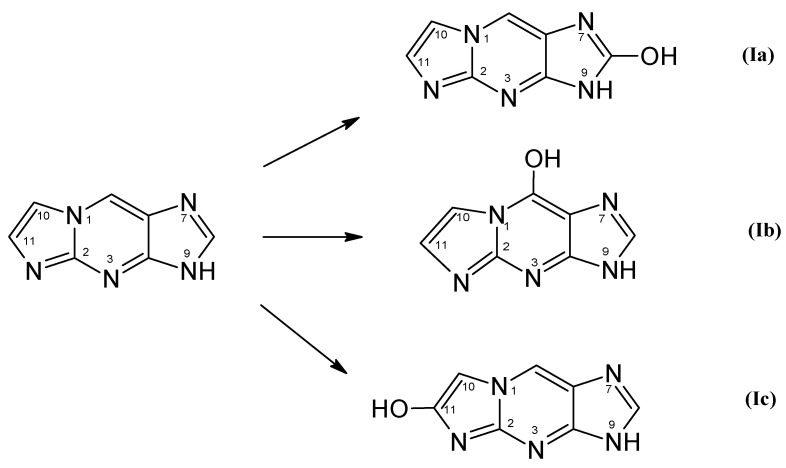
Structure and possible primary products of enzymatic oxidation of 1,N^2^-ε2APu (**I**). Note that these products may exist in aqueous solutions in the form of enol keto tautomer, as well as in various ionic forms.

We therefore attempted to co-crystallize the new substrates with purified enzymes: PNP from *E. coli* and XO from bovine milk. This was partially successful, as complexes of the wild-type PNP and its D204N mutant were obtained with only the “non-linear” substrate (N^2^,3-ε2APu, **II**) bound. The values of dissociation constants estimated from the ITC experiments may explain these results, as in the case of the “linear” derivative (1,N^2^-ε2APu, **I**), K_d_ is in the millimolar range, while that for the N^2^,3-ε2APu (**II**) is 36.16 μM (with the asymmetric confidence intervals of 29.33–45.89 μM). Previously, we concluded that the predominant ribosylation site is N2 [17], rather than N9 typical for the natural substrates. From the X-ray diffraction on a protein crystal, we observe N^2^,3-ε2APu (**II**) in the active site of the enzyme, but its orientation cannot be determined unambiguously as the electron density blob is symmetric (Scheme 3, Section 2.1.3).

Due to the symmetry of the electron density assigned to the ligand, we are unable to determine exactly how the ligand binds to the Asp204, which is the key catalytic residue. Depending on the arrangement of the molecule, Asp204 can interact with N7 (Scheme 3c), N^2^ (Scheme 3d), or N9 (Scheme 3a), while in the last orientation, a carbon atom is present against Asp204 (Scheme 3b), and formation of a strong hydrogen bond is not possible. Accordingly, ribosylation would occur at positions N9 (Scheme 3c), N7 (Scheme 3a), and N^2^ (Scheme 3b), or no ribosylation would be observed (Scheme 3d). Comparison with the data from the enzymatic synthesis conducted in solution suggests that only variant (3b) is likely realized, but still, the detailed reaction pathway is unclear. Alternative reaction schemes, not involving Asp204, were proposed for some purine analogs a few years ago [47] and we cannot exclude their role in this non-typical ribosylation.

In the case of 1,N^2^-ε2APu (**I**) molecule, in subunits B of both complexes (WT-PNP and D204N-PNP), in the place usually occupied by the purine ring, there is only an array of small electron density blobs fitting well with a few water molecules. In subunits A and C (forming one dimer), the blobs are too small to fit in a 1,N^2^-ε2APu molecule (Figure 2). We therefore conclude that the affinity of PNP to (**I**) is probably low and that the ligand binds only in a small number of protein molecules in the crystal. This was confirmed by the isothermal titration calorimetric data for (**I**), K_d_ is higher than 3.5 mM, while for (**II**), K_d_ = 36.16 μM (with nonsymmetrical confidence intervals 29.33–45.89 μM); hence, binding is 100 times better than that of 1,N^2^-ε2APu.

Attempts to obtain crystals of xanthine oxidase complexes with 1,N^2^-ε2APu, as well as with N^2^,3-ε2Apu, were not successful. A possible explanation was proposed by Hille [27], who pointed to a marked genetic heterogeneity in the bovine population as a source of this problem (finally successfully resolved).

For both compounds, isothermal calorimetric titration data are complicated, but allow estimation of the dissociation constant, K_d_. In both cases, it is less than 1 μM, hence, it is difficult to decide which compound is a better marker.

Direct kinetic experiments with purified bovine XO (Section 2.2.3, Section 2.2.4, Section 2.2.5, Section 2.2.6 and Section 2.2.7) and two etheno-2AP isomers indicated that in both cases the oxidation products are strongly fluorescent, but (**I**) is definitely a better substrate, with reaction rates only slightly lower than those of the natural substrates like xanthine (Figure 9). Moreover, the emission spectrum of the oxidation product of (**II**) does not differ markedly from that of the substrate (see Supplementary Data S4), making any quantitative determination of activity difficult. By contrast, oxidation of (**I**) leads to a reasonable (~10-fold) increase in fluorescence near 400 nm (Figure 7).

Oxidizing enzymes like XO usually do not lead to highly fluorescent reaction products and known fluorimetric assays for XO are rare and rather laborious [48,49], so this simple assay may be useful, as it offers potentially high throughput.

The kinetics of XO catalyzed reaction is non-classical, showing the apparent substrate inhibition [44], especially evident at pH 7, and somewhat less marked at pH 9 (see Figure 9a). For this reason, it is difficult to isolate the fluorescent product in quantities sufficient for crystallization, hence, its identification as (**Ia**) remains uncertain.

We have shown that the fluorimetric assay for XO based on (**I**) as a substrate may be applied to milk and dairy food analysis, which is difficult for spectrophotometry due to milk turbidity. But even 100-fold diluted bovine milk shows very good XO activity, which decays upon thermal treatment (Figure 10) and is sensitive to XO inhibitors or competitors (Figure 11). The sensitivity of this assay is then comparable to the older resazurin–resorufin assay [50,51], based on fluorimetric detection of the produced hydrogen peroxide. Optimal conditions for the milk assay are pH 9, initial concentration of the substrate ca. 40–50 μM, and temperature ~30 °C. Fluorescence increase should be monitored at 400 nm with excitation at 310–320 nm.

Potential clinical applications of this method (e.g., determination of XO activity in blood serum, plasma, or other body fluids, which may prove useful for diagnostics of liver and cardiovascular diseases [30,32]) should be examined.

Other applications of the presented assay are possible but require a thorough examination of a variety of XO or XDH isoforms, as reported in various organisms [52]. Extension to the intracellular xanthine dehydrogenase would be interesting, but possible interference by other oxidizing enzymes like aldehyde oxidase and cytochromes remains unknown.

## 4. Materials and Methods

### 4.1. Enzymes, Chemicals, and Food Samples

Commercially available xanthine oxidase (XO, Sigma grade IV, suspension in aqueous NH_4_Cl, ~0.3 U/mL, 11 mg protein per mL) from bovine milk was used to screen the tri-cyclic nucleobase analogs (see Scheme 1) as possible XO substrates (or inhibitors). The enzyme suspension was diluted 10-fold in 5 mM phosphate buffer, pH 7, containing 0.5 mM EDTA, and stored in a refrigerator. This diluted enzyme was stable for more than 1 month. For individual screening of the potential substrates, the stock solution was further diluted 100–500-fold.

Recombinant wild-type and D204N mutant of *E. coli* PNP and wild-type calf spleen PNP were expressed in *E. coli* and purified according to the procedures described earlier [53,54].

The syntheses of 1,N^2^-ε2APu (**I**) and of the isomeric N^2^,3-ε2APu (**II**) were described previously, and the purity of the compounds was confirmed by HPLC and NMR analysis [17]. Mass spectra of both compounds and NMR data are presented in Supplementary Material S5 (Table S1 and Figure S1). Xanthine, oxipurinol, and 2-aminopurine (2APu), as well as chloroacetic aldehyde (50% aqueous solution), were from Sigma-Aldrich. All buffer constituents were of analytical grade and did not contain fluorescent contaminants.

Purification of XO substrates by re-crystallization from water, and their subsequent enzymatic oxidation, was followed by chromatographic separation by HPLC (Shimadzu Corp.), with UV-absorbance and fluorescence detectors. Semi-preparative C-18 (Kromasil) column was eluted by 10 mM phosphate buffer-methanol gradient (typically 3–8%). Analytical HPLC was performed analogously, with a small-size analytical column and gradient extended to 1–25%.

Milk samples were from Mlekovita (Wysokie Mazowieckie, Poland), and pork liver from commercial food stores. Milk samples were analyzed within 24 h of milking (unprocessed sample) or at least 7 days prior to the expiration date (commercial milk samples).

The liver homogenate was prepared as described in Appendix B.

### 4.2. Crystallization, Data Collection and Structure Determination

Crystals of *E. coli* PNP in a ternary complex with phosphate (sulfate) and the chosen ligand (1,N^2^-ε2APu or N^2^,3-ε2APu) were obtained by the hanging-drop vapor diffusion method. For crystallization, storage buffer (50 mM Tris/HCl buffer pH 7.6) was exchanged for a 10 mM citrate solution, pH 7.0. Drops with the WT *E. coli* PNP contained 60 mg/mL (2.3 mM) of the protein, whereas those containing D204N mutant were 40 mg/mL (1.6 mM). Ligand molar concentration was at least 2 times higher than the protein molar concentration (5 mM and 3 mM of each ligand for WT-PNP and D204N-PNP, respectively). The drops were set up at 18 °C by mixing 1.1 μL of protein–ligand complex in a 1:1 ratio with 1.1 μL of the reservoir buffer (100 mM citrate buffer pH 5.2 and 5.4, 14–20% ammonium sulfate) and allowed to equilibrate against the reservoir buffer. The crystals with half of the hexamer in the asymmetric unit appeared as hexagonal rods (space group P6_1_22) after 1–2 weeks and remained stable for long periods. Crystals were soaked in cryoprotectant solution containing 30% glycerol prior to flash freezing in liquid nitrogen.

Data were collected at beamline P11 operated by EMBL Hamburg at the PETRA III storage ring (DESY, Hamburg, Germany), courtesy of Elżbieta Nowak, Laboratory of Protein Structure, International Institute of Molecular and Cell Biology, Warsaw. Data were obtained by rotation with Δφ = 0.1°, with a single crystal. Data collection and refinement parameters for the structures are summarized in Appendix C, Table A1. The data were integrated using XDS (version Feb 5, 2021) software [55]. All structures were solved by the molecular replacement using the ccp4/phaser (version 2.8.3) software [56] and structure 4TS9 of *E. coli* PNP [57] as a model. The structures were refined with ccp4/Refmac [58] and COOT [59], and the finalization of structures for deposition was performed with phenix.refine [60]. Composite omit electron density maps (mFo-DFc) were calculated with phenix.composite_omit_map software from the Phenix Suite version 1.21.1.5286 [60]. Figures of crystallographic structures were prepared with PyMOL (Schrödinger, New York, NY, USA).

For XO crystallization, a commercially available protein (X4500, Sigma-Aldrich, Merck KGaA, Darmstadt, Germany) was used. Since it turned out that the sample was not electrophoretically pure, it was further purified by size exclusion chromatography (SEC) using the Superdex 200 (Cytiva) column (see Supplementary Data S3). Fractions of the highest purity and activity were used for setting crystals. Crystallization was carried out by the hanging- and sitting-drop vapor diffusion methods, at 18 °C. Droplets were prepared by mixing 1.0–10 μL of the apo-protein or a complex with one of the ligands with crystallization solutions at various proportions. The tested conditions were based on data available in the literature [39,40], and were as follows: 7.5–40 mg/mL XO, 100 mM potassium phosphate buffer pH 6.0–8.0, PEG 4000 4–15% *w*/*v*, glycerol 30%, PEG 8000 10–15% *w*/*v*, crystallization solution contained also 0.2 mM EDTA, 1 mM sodium salicylate, and 5 mM DTT. Small, irregular, not well-diffracting crystals were obtained only for the apo-protein in conditions with 12–14% PEG 8000, pH 6.5.

### 4.3. Enzyme Kinetics, Inhibition and Competition with Xanthine

Spectral measurements were applied to perform standard control of the enzyme activity and to follow the kinetics of the enzymatic reactions. Steady-state emission spectra were measured on a Varian Eclipse instrument (Varian-Australia), with a typical spectral resolution of 2.5 or 5 nm, and UV absorption spectral measurements were performed on a Cary 5000 (Varian) thermostated spectrophotometer. Emission spectra determinations and fluorimetric assays were typically performed in semi-micro cuvettes with a pathlength of 4 mm to diminish the inner-filter effect. Quantum yields and fluorescence decay times of the substrates and products of the enzymatic reactions were measured as described previously [17].

Kinetic analysis of enzymatic processes was performed according to standard methods, as described in our previous papers [15,16,17,24]. Substrate concentrations and reaction rates were determined spectrophotometrically using known extinction coefficients (Table 1). In fluorimetric assays, rates were calculated using the following Formula (1):

(1) $$v=\frac{dc}{dt}=\frac{dF}{dt}\cdot \frac{C_{0}}{∆F}$$

where *C*_0_ is the initial concentration of the substrate, Δ*F* is the total fluorescence change during the reaction, and *dF/dt* is the fluorescence change in time. For slow reactions, Δ*F* was determined after the addition of purified XO in an amount sufficient to complete the oxidation of the substrate in the sample or measured independently.

### 4.4. Calorimetric Titrations

For PNP from *E. coli* and milk XO, the measurements were carried out with the ITC200 (MicroCal/Malvern Panalytical, Malvern, United Kingdom, ) at 25 °C, with 22 injections per titration, in 50 mM phosphate buffer pH 7.6 and 50 mM potassium phosphate buffer with 1 mM TCEP pH 8.0, respectively. Samples of protein were dialyzed into the appropriate buffer and filtrated with 22 μm pore filters.

Commercially available XO (X4500, X4875, Sigma-Aldrich, Merck KGaA, Darmstadt, Germany and 38418, Serva Electrophoresis, Heidelberg, Germany) in the form of ammonium sulfate suspension was used. Protein suspension was centrifuged (10 min, 10,000 RPM), and the pellet was re-suspended in 50 mM potassium phosphate buffer with 1 mM TCEP pH 8.0.

In the case of 1,N^2^-ε2APu (**I**), the powder was dissolved in the same buffer in which the protein was dialyzed, while in the case of N^2^,3-ε2APu (**II**), the stock of 9.5 mM concentration was diluted with a buffer the protein was dialyzed in. All samples were degassed for at least 0.5 h prior to measurement. Thermograms were integrated with the NITPIC (version 1.3.0) software [61]. For each pair of enzyme–ligand, the experiments were performed in series, from two to four titrations, with varying protein concentration (thus varying Wiseman coefficients) and varying final ligand/protein concentration ratio, and were analyzed globally with the ITCsy (version 1a) software using the software-implemented models [43]. The simplest, one-binding-site model of association, P + L ↔ PL, and a more complicated model, assuming the presence of two unknot equivalent binding sites, P + L ↔ PL + L ↔ PLL, were tested. For each experiment, a ligand-buffer titration was performed and subtracted from the ligand–protein titration.

## Figures and Tables

**Table 1:** Fluorescence properties of the tri-cyclic nucleoside analogs (etheno derivatives) and their ribosides (neutral forms, compiled from refs. [17,25]).

Compound	Nucleobase		Riboside		Refs.
(Nucleobase)	$\lambda_{em}^{max}$[nm]	ϕ	$\lambda_{em}^{max}$[nm]	ϕ	
1,N^2^-ε2APu	472	0.18 *	463 ^a^	0.14 ^a^	[17]
			406 ^b^	~0.7 ^b^	[25]
N^2^,3-ε2APu	406	0.73	nd ^a^	nd ^a^	[17]
			357 ^b^	0.29 ^b^	

* Maximum and yield of fluorescence dependent on excitation wavelength; nd—no data. ^a^ N^9^-riboside; ^b^ N^2^-riboside.

**Table 2:** Spectral data for the tri-cyclic substrates of XO, 1,N^2^-ε2APu (**I**), and N^2^,3-ε2APu (**II**), and the products of their oxidation. Data from [16,17] and this work. Fluorescence was excited at 280 nm. Excitation-dependent data are marked with an asterisk (*).

Compound	pK_a_	Form (pH)	UV Absorption λ_max_ (nm) (ε_max_)	Fluorescence λ_max_ (nm)	Fluorescence Quantum Yield ϕ	Fluorescence Decay Time (ns)
1,N^2^-ε2APu (**I**)	5.6; 8.2	neutral (6.8)	348 (2560)	465	0.17 ± 0.02 *	6.9; 10.3
		anion (11.0)	367 (3500)	473	~0.4	nd
Main oxidation product (**Ia**)	5.65; 8.5	neutral (7.1)	295 (~6000)	448	0.30 ± 0.03 *	nd
		anion (10.5)	298 (~5500)	450	0.34 ± 0.03	nd
(**Ib**)	~2; 9.5	neutral (7.0)	287 (9700)	-	nf	-
		anion (11.5)	303 (7200)	400	<0.01	nd
N^2^,3-ε2APu (**II**)	~5.0; 9.5	neutral (7.1)	315 (5600)	406	0.73 ± 0.05	3.8; 8.5
Main oxidation product of N^2^,3-ε2APu	nd	neutral (7.0)	322 (~5000)	385	>0.5	nd

nd—no data; nf—no fluorescence detected.

## Data Availability

Structures described in this paper are deposited in PDB and accession numbers are 9FPE for complex WT PNP with N^2^,3-ε2APu and 9FXE for complex D204N-PNP with N^2^,3-ε2APu. Structures of both enzyme variants with 1,N^2^-ε2APu, and all other data are available upon reasonable request.

## Supplementary Materials

The following supporting information can be downloaded at: https://www.mdpi.com/article/10.3390/ijms251910426/s1.

## Appendix A

### Linearity of the Proposed Assay of XO

Linearity of the fluorimetric XO assay (in logarithmic scale) for enzyme concentrations 10^−6^ to 10^−4^ U/mL was analyzed (Figure A1). For higher enzyme concentrations, the reaction is very rapid; therefore, milk samples must be diluted at least 100-fold to be analyzed properly (see above, Section 2.2.3). For optimal substrate concentrations (5–50 μM), the reproducibility of the procedure is better than 5% (based on three repeats).

## Appendix B

### Substrate **I** Oxidation by Liver Homogenate

To assess the degree of specificity of the proposed assay, we decided to check if the liver homogenate, containing a mixture of various oxidizing enzymes, including cytochromes and aldehyde oxidase, known to be active for N-heterocycles [62,63], can potentially interfere with the fluorimetric procedure involving (**I**) as a substrate.

As can be seen in Figure A2, the substrate (**I**) is rapidly (t_1/2_~5 min) degraded by the 100-fold diluted pork liver homogenate (the concentrated homogenate contained ca. 0.5 g chopped pork liver per 2 mL of buffer). However, no fluorescence increase was recorded during this reaction, and the reaction rate was virtually insensitive to 15 μM oxipurinol. Most likely, neither XO nor its intramolecular variant (xanthine dehydrogenase) is involved in this reaction. Spectral analyses show that the main product was, in this case, the non-fluorescent 1,N^2^-etheno-guanine (**Ib**), while oxidation by XO most likely leads to the fluorescent product (**Ia**).

We therefore conclude that the fluorimetric procedure is fairly selective towards XO and minor amounts of other oxidizing enzymes do not interfere with the proposed XO fluorimetric assay, particularly in bovine milk samples. Potential clinical applications of this method (e.g., determination of XO activity in blood serum, plasma, or other body fluids) should be examined.

## Appendix C

### Data Collection and Refinement Parameters for the Crystal Structures of WT PNP and D204N-PNP with N^2^,3-ε2APu

**Table A1:** Data collection and refinement parameters for the structures obtained for WT PNP and D204N-PNP complexes with N^2^,3-ε2APu. The structures were solved by the molecular replacement using the ccp4/phaser software and the structure 4TS9 of *E. coli* PNP as a model.

PDB ID	WT	D204N
	9FPE	9FXE
Data collection		
Space group	P 6_1_ 2 2	P 6_1_ 2 2
Cell dimensions		
a, b, c (Å)	120.28, 120.28, 239.41	120.53, 120.53, 239.21
α, β, γ (°)	90.0 90.0 120.0	90.0 90.0 120.0
Resolution (Å)	48.03–1.60 (1.63–1.60) *	47.9–2.12 (2.196–2.12)
No. of observations	5,469,130 (275,823)	2,391,009 (201,004)
No. unique reflections	134,561(6595)	59,360 (5825)
Rmerge	0.170 (3.915)	0.29 (3.43)
Rmeas	0.172 (3.963)	0.299 (3.481)
Rpim	0.037 (0.831)	0.0455 (0.587)
CC1/2	1.000 (0.840)	0.999 (0.589)
Mean I/σ(I)	16.8 (1.3)	15.24 (1.15)
Completeness (%)	100.0 (100.0)	97.46 (100.00)
Multiplicity	40.6 (34.7)	40.3 (34.5)
Wilson B-factor	23.33	38.38
Refinement		
Resolution (Å)	48.03–1.6 (1.657–1.6)	47.9–2.12 (2.196–2.12)
R_work_/R_free_	0.18/0.20	0.21/0.23
No. reflections all/free	134,438/6675	57,889/2794
Protein residues	711	711
Number of nonhydrogen atoms		
protein	5406	5391
ligands (N^2^,3-ε2APu, SO_4_, glycerol)	78	51
water	504	180
Ramachandran		
favored (%)	97.73	96.03
allowed (%)	2.27	3.40
outliers (%)	0.00	0.57
Clashscore	1.55	2.30
RMSD bonds (Å)	0.007	0.007
RMSD angles (°)	0.95	0.88

* high-resolution bin in parentheses.
