# Supplementary material for: Interaction of Tri-Cyclic Nucleobase Analogs with Enzymes of Purine Metabolism: Xanthine Oxidase and Purine Nucleoside Phosphorylase
Source: Int J Mol Sci. 2024 Sep 27;25(19):10426. doi: 10.3390/ijms251910426 (PMC11477426; doi:10.3390/ijms251910426)

Interaction of the tri-cyclic nucleobase analogs with enzymes of purine metabolism: xanthine oxidase and purine nucleoside phosphorylase

Alicja Stachelska-Wierzchowska[1], Marta Narczyk[2], Jacek Wierzchowski[1], Agnieszka Bzowska[2*] and Beata Wielgus-Kutrowska[2*]

SUPPLEMENTARY DATA

S1 Isothermal Titration Calorimetry data for PNP-WT binding with 1,N^2-ε2APu

ITC titrations data for binding of the wild type PNP with 1,N^2-ε2APu. Titration was performed with 40.19 μM of protein in the calorimeter cell, at 25 °C, in 50 mM potassium phosphate buffer with 1 mM TCEP pH 8.0.
Below are shown thermograms of the protein-ligand titration (violet) and of the buffer-ligand titration (red). Titration of the buffer with the ligand was performed with shorter time between injections (200 s for protein-ligad vs.150 s for buffer-ligand).

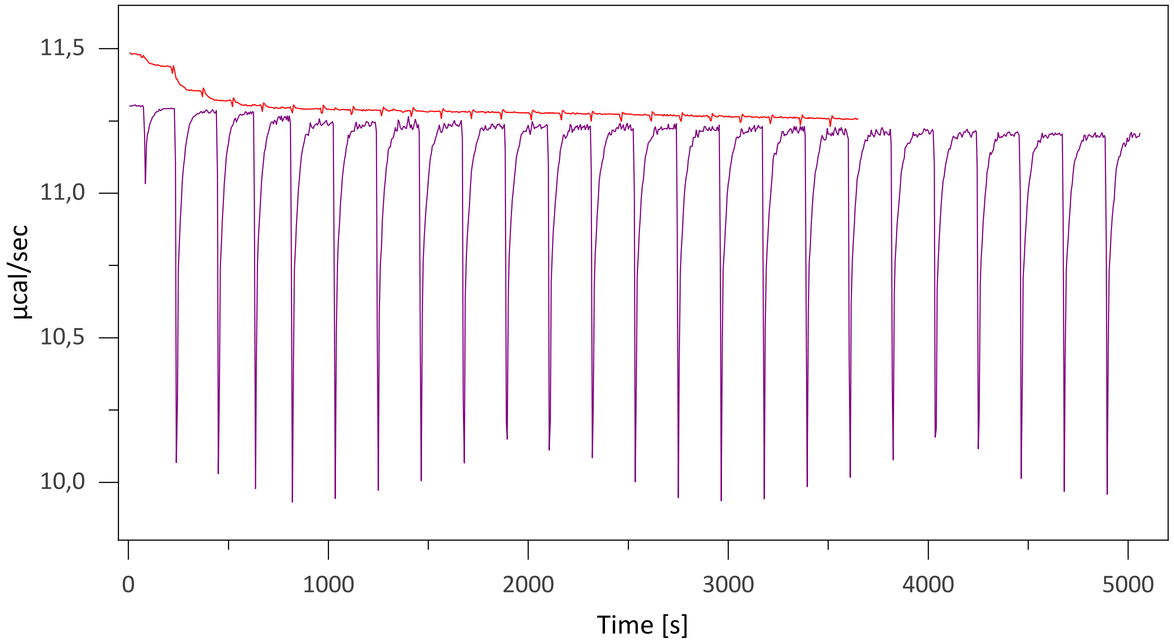

Below the integrated isotherms of the thermogram shown above are presented.
Protein-ligand titration (violet) and buffer-ligand titration (red).

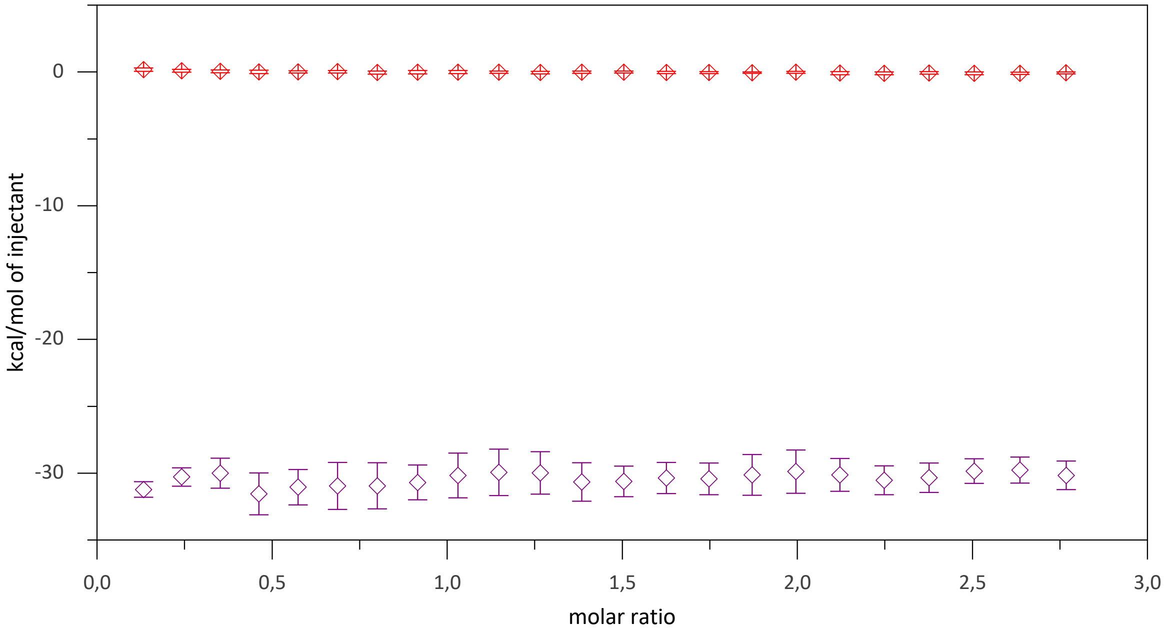

S2. Milk-catalysed oxidation reaction of 1,N²-ε2APu (I) followed by HPLC

Below is HPLC elution profile (absorbance monitored at 280 nm) of the oxidation reaction of ~80 μM substrate, 1,N²-ε2APu (**I**) catalyzed by 100-fold diluted milk. The reaction was carried out in 20 mM phosphate buffer, pH 7. Solid blue line is measured prior to the reaction, dashed green line – after 1 hr of the reaction, dotted red line – after 4 hrs of the reaction.

Note that there is no indication of any consecutive reaction of the product (**Ia**).

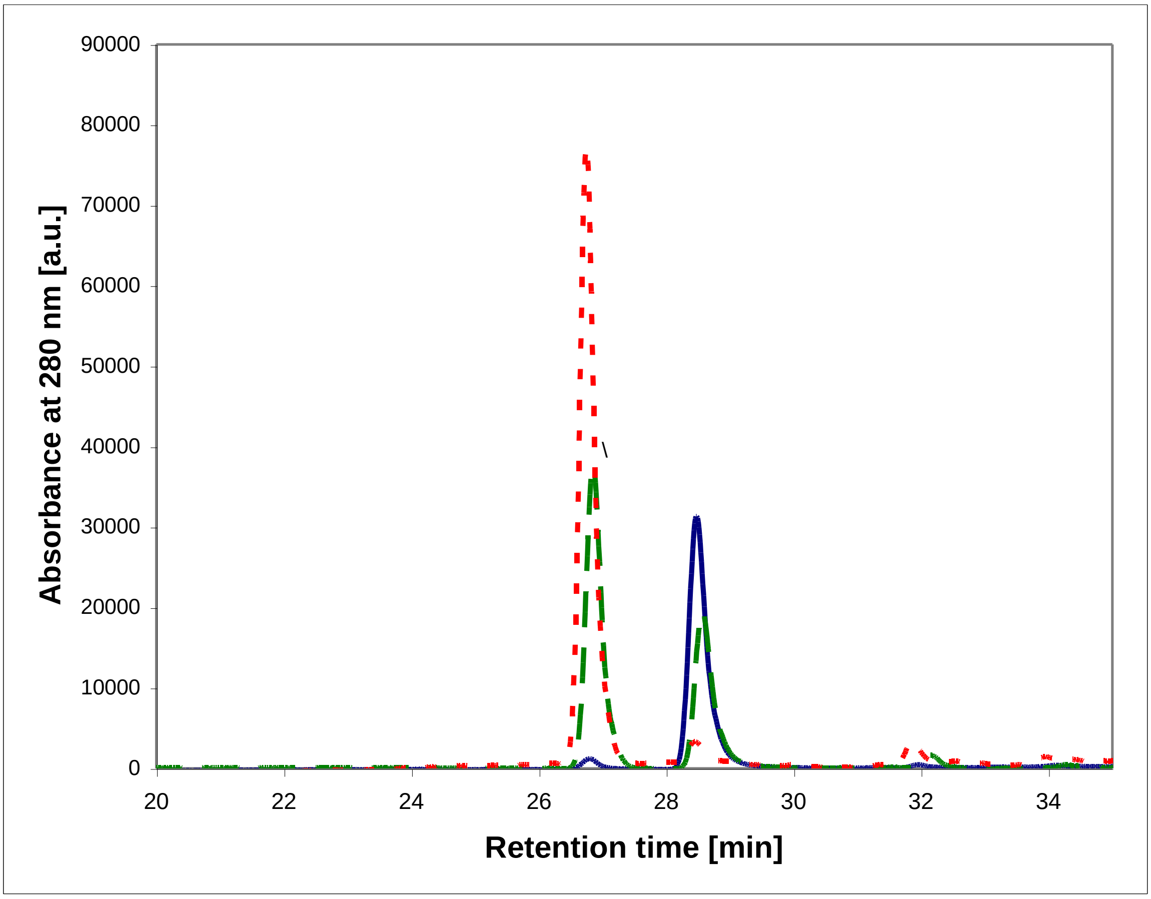

S3 Example of SEC chromatogram obtained during purification of the commercially available xanthine oxidase (XO)

Xanthine oxidase from bovine milk (XO, Sigma grade IV, suspension in aqueous NH_4Cl, ~0.3 U/mL, 11 mg protein per mL) purified by size exclusion chromatography (SEC) using the Superdex 200 (Cytiva) column. Absorption at 280 nm (green) and enzymatic oxidation activity (orange) profiles are overlaid. The elution buffer was 100 mM potassium phosphate buffer + 0.2 mM EDTA + 1 mM sodium salicylate, pH 8.5, the elution rate was 0.8 mL/min, volume of one fraction was 1.3 mL. High noise observed in the absorption 280 nm profile is due to the presence of EDTA in the buffer. Fractions were combined together into three samples XO1, XO2, XO3 as shown by arrows. Only sample XO1 was used for setting crystals.

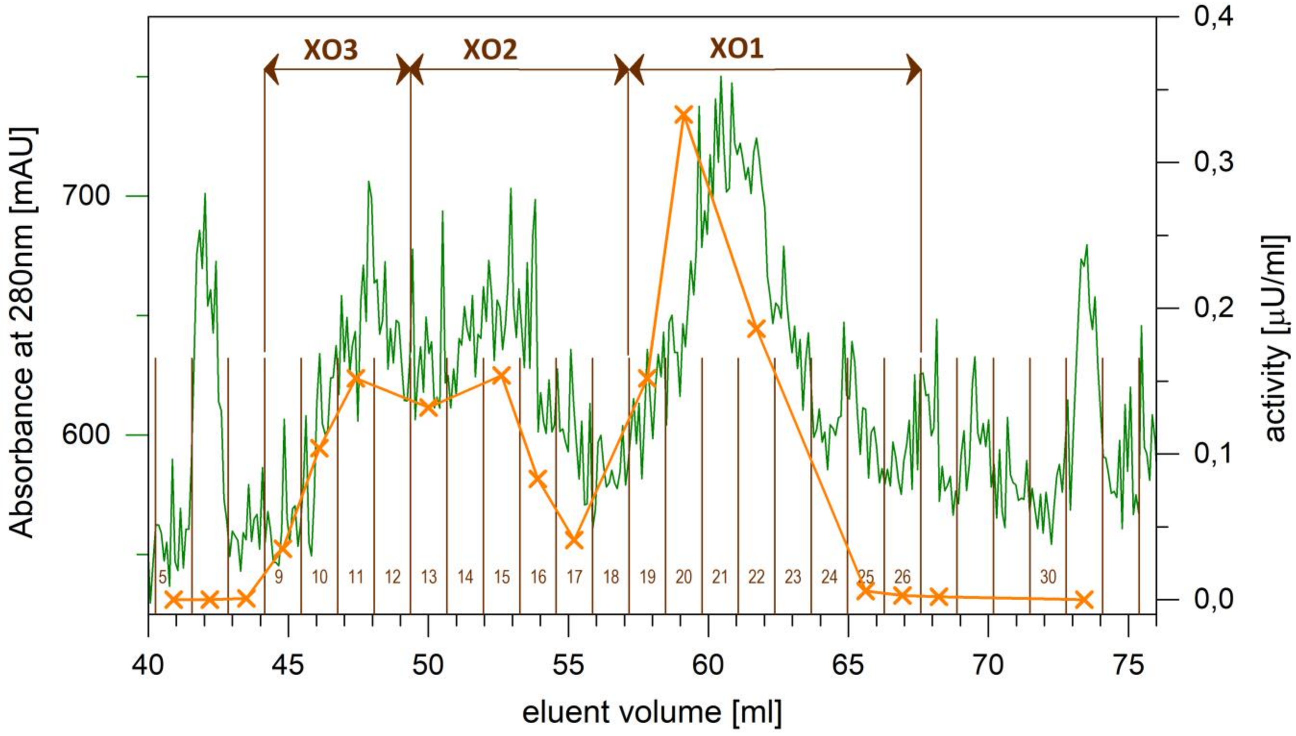

S4. Enzymatic oxidation of substrate II (N^2,3-etheno-2-aminopurine).

Below are shown spectral changes observed during the enzymatic oxidation of (**II**) using bovine xanthine oxidase: (upper panel, fluorescence, lower panel, UV absorption). Substrate concentrations were 40 μM for UV absorption and 5 μM for fluorescence. Violet lines – reaction start, red lines – reaction end. Reaction conditions as in the Figure 7.

Note that the enzymatic oxidation of (**II**) is at least 10-fold slower than that obtained for (**I**).

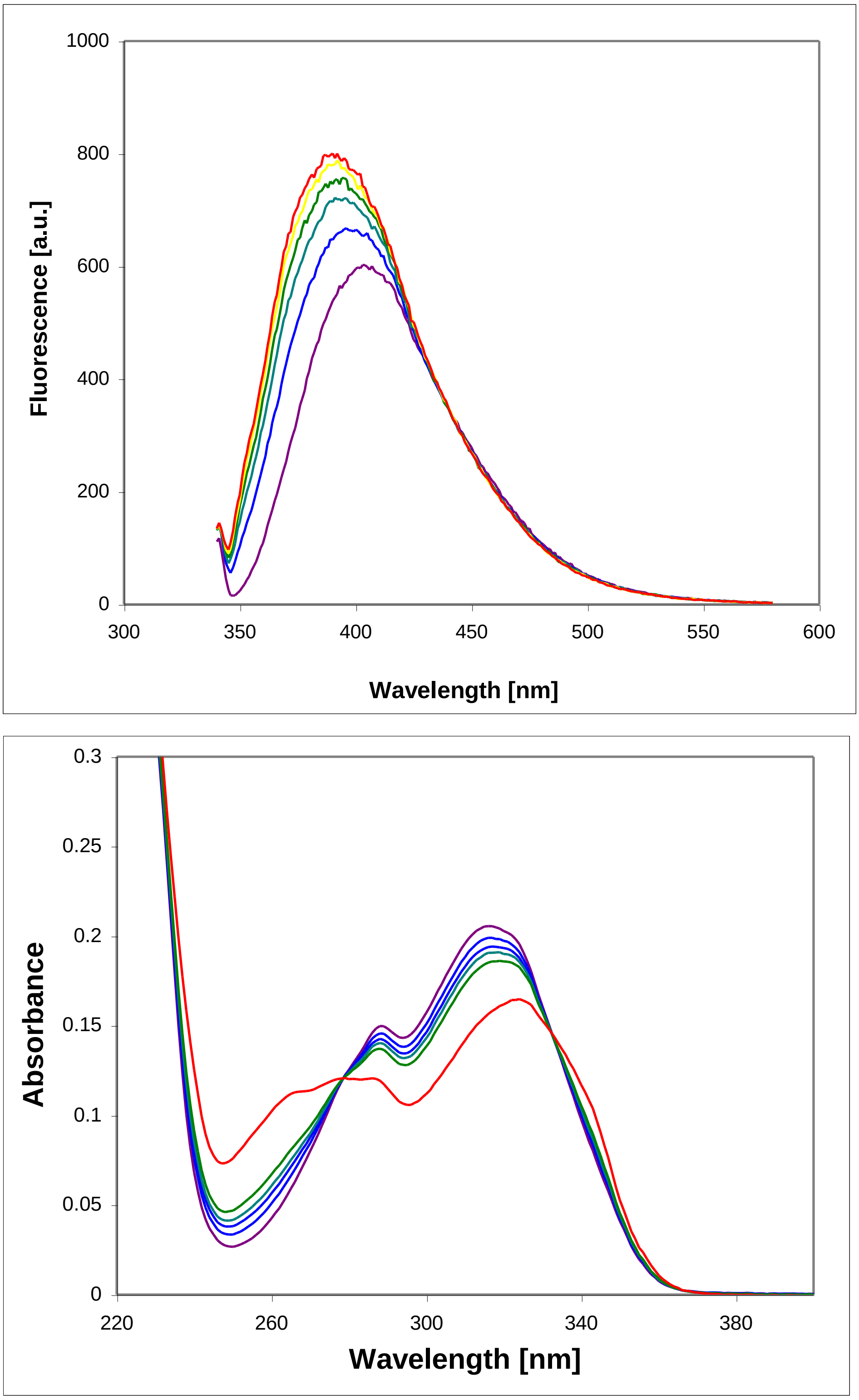

S5. NMR data and mass spectra of the substrates I (1,N²-ε2APu, upper panel) and II (N²,3-ε2APu, lower panel).

Table S1. NMR data for I and II in D_2O (neutral forms).

**Compound/concentration**	**H6**	**H8**	**H10/11**	**H10/11**
**I**/ 4 mM	9.23s	8.49s	7.87d	7.68d
**II**/ 2.5 mM	8.97s	8.38s	8.12d	7.80d

s-singlet, d-doublet.

Figure S1. Mass spectra of compounds I (upper panel) and II (lower panel).

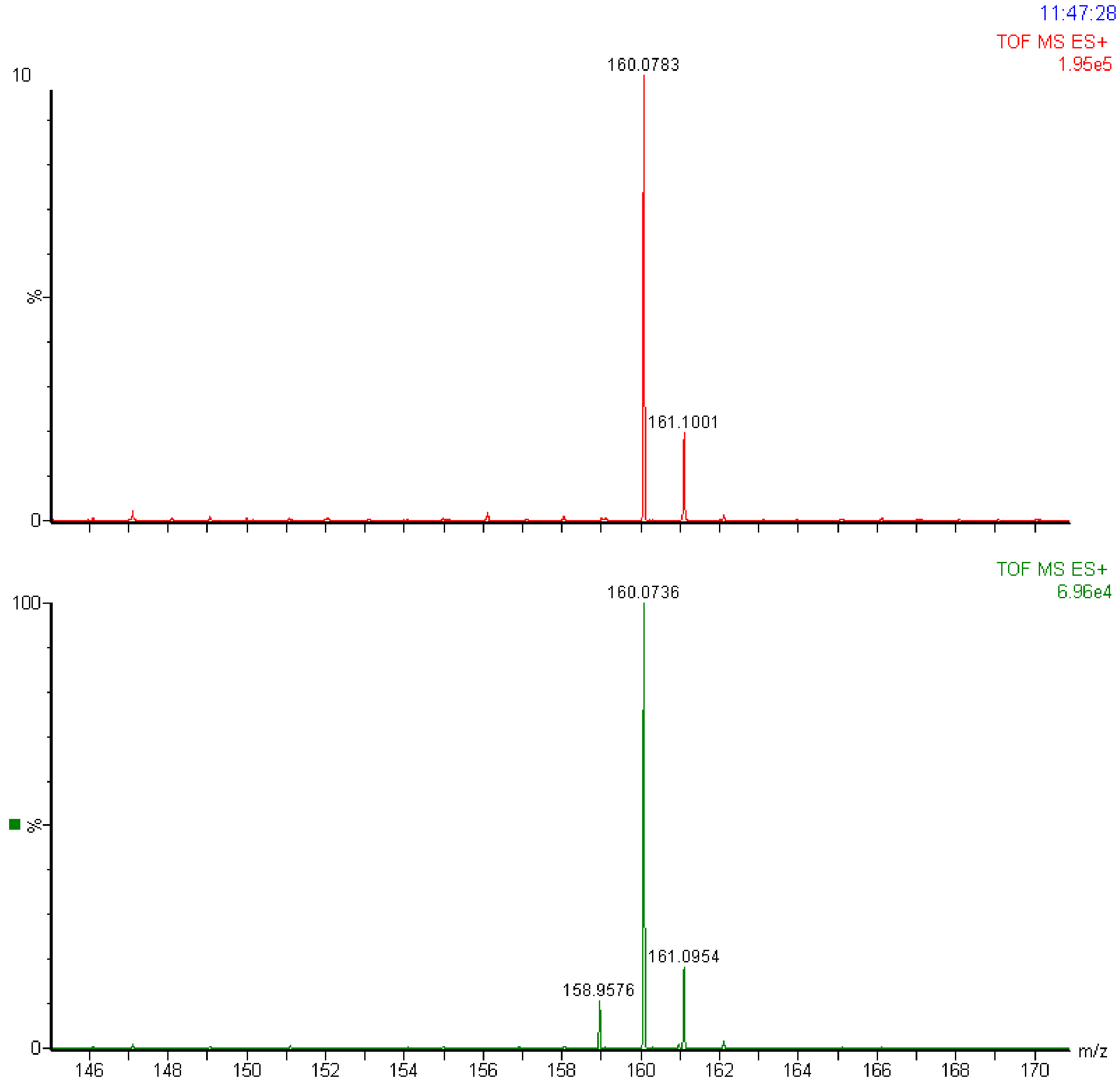

Supplement: Supplementary file 1 [file ijms-25-10426-s001.zip › ijms-3235915-supplementary.pdf]
